# On Oscillations in the External Electrical Potential of Sea Urchins

**DOI:** 10.1021/acsomega.4c10277

**Published:** 2025-01-08

**Authors:** Panagiotis Mougkogiannis, Andrew Adamatzky

**Affiliations:** Unconventional Computing Laboratory, University of the West of England, Coldharbour Ln, Stoke Gifford, Bristol BS16 1QY, U.K.

## Abstract

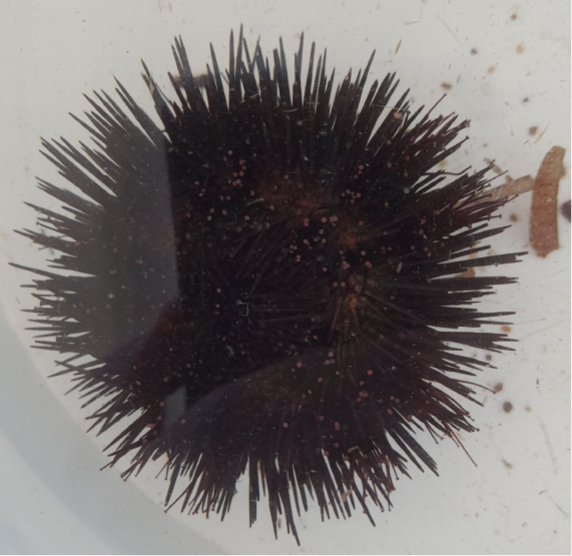

Sea urchins display complex bioelectric activity patterns, even with their decentralized nervous system. Electrophysiological recordings showed distinct spiking patterns. The baseline potential was about 8.80 mV. It had transient spikes with amplitudes up to 21.05 mV. We observed many types of depolarization events. They included burst-like activity and prolonged state fluctuations lasting several seconds. Frequency domain analysis showed a power-law behavior. It had a scaling exponent of 6.21 ± 0.06, indicating critical dynamics. The analysis showed potential variations between 3.69 and 21.05 mV. The oscillation periods ranged from 4 to 3102 s. The varied timing of bioelectric signals suggests that these organisms can process information. This challenges traditional views of neural computation in simpler animals. These findings provide quantitative insights into the complex signaling mechanisms of the sea urchin’s distributed nervous system.

## Introduction

Biocomputation, a field that examines the principles of biological systems-based information processing, has seen significant development in recent years as scientists seek to discover innovative solutions for complex computational problems.^1^ This emerging field takes inspiration from the various information processing systems seen in nature, including the complex brain networks of vertebrates and the chemical signaling pathways of single-celled organisms.^2^ As our understanding of biological information processing grows, the possibility of generating groundbreaking computing models that could transform biological artificial intelligence, robotics, and environmental monitoring technologies also increases.

The examination of sea urchins has a rich historical background that can be traced back to ancient times. Aristotle, during the fourth century BC, was one of the earliest individuals to provide a detailed account of the anatomical structure, behavior, and wide range of species within this group. He notably used the analogy of a lantern to describe the sea urchin’s test, which led to the term “Aristotle’s lantern” being mistakenly applied to the jaw apparatus by later zoologists.^3^

There is a highly promising field in biocomputation that focuses on the study of spiking neural networks (SNNs). These networks aim to closely replicate the information processing abilities of biological neurons, going beyond what traditional artificial neural networks can achieve.^4^ SNNs function based on discrete spikes or action potentials, which resemble the patterns found in biological nervous systems, instead of using continuous activation functions.^5^ This approach not only presents a neural computation model that is in accordance with biological principles but also has the potential to create computing systems that are more energy-efficient and adaptable.^6^

There has been a significant amount of research dedicated to studying SNNs in vertebrate nervous systems. However, there is now a rising curiosity about investigating the computational capabilities of simpler organisms.^7^ This change in perspective is motivated by the realization that even seemingly basic organisms may have advanced information processing abilities that could be valuable for developing new biocomputation methods. Among these organisms that have not been extensively studied, sea urchins have become a fascinating model system for exploring spike-based biocomputation.

Sea urchins, which belong to the phylum Echinodermata, have been extensively studied in the field of developmental biology. However, their potential in neuroscience research has been relatively overlooked.^8^ Recent research has revealed a fascinating discovery about these marine invertebrates: they have a sophisticated electrical signaling system that resembles the neural spikes found in more advanced organisms.^9−12^ The identification of these electrical potential spikes in sea urchins has opened new possibilities to investigate information processing that resembles neural activity in a system that is evolutionarily distinct from vertebrates and structurally less complex than conventional neuroscience models.

Understanding sea urchin electrical signaling holds great importance for various reasons. First, it provides valuable information on the evolutionary origins of neural-like information processing, which could help us better understand the fundamental principles behind computation in biological systems.^13^ Furthermore, the anatomy of sea urchins is relatively simple compared to vertebrate nervous systems, making it a more practical model for studying spike-based information processing.^14^ In fact, the remarkable ways in which sea urchins have adapted to their marine environment may hold valuable information on new computational strategies, which could spark novel concepts for biocomputation.^15^

Echinoderms are unique organisms with special features that set them apart from other groups. They have a water vascular system and a calcium carbonate endoskeleton, called the stereom. Most echinoderms start off as larvae and go through a complex metamorphosis to become adults. They can reproduce sexually or asexually. Echinoderm larvae are usually free-living and planktonic and have diverse shapes that sometimes resemble hemichordate larvae. When they become adults, echinoderms live on the ocean floor and have radial symmetry and a pentameric structure. Their internal anatomy is complex, with calcium carbonate skeletons supported by collagen ligaments. Echinoderms develop from bilaterally symmetrical larvae but mature into animals with pentaradial symmetry. This unique combination of features places them in an interesting evolutionary context as invertebrate deuterostomes closely related to vertebrates.^16^

This study investigates the possibility of neural-like information processing in sea urchins, specifically examining the characteristics and functional significance of their electrical potential spikes. We have three main research objectives:

1 To analyze the patterns of electrical potential spikes in sea urchins in response to different environmental stimuli.
2 To explore the possible impact of these spikes on information processing and decision-making behaviors in sea urchins.
3 To explore the potential of sea urchin spike-based signaling in the development of innovative biocomputation frameworks.

This research is unique in its interdisciplinary approach, bringing together the fields of marine biology, neuroscience, and computer science. Through the use of advanced electrophysiological techniques and computational modeling, our research focuses on exploring the unexplored potential of information processing in sea urchins by studying their electrical signaling. In addition, our research aims to demonstrate the potential of sea urchins as a valuable model system for biocomputation research. This could lead to the exploration of new possibilities for bioinspired computing architectures. In the upcoming sections, we will outline our approach, highlight our major findings, and engage in an in-depth discussion regarding the implications of our research for the field of biological information processing and the future of biocomputation. With this research, our aim is to question traditional ideas about neural-like computation and spark new concepts in the realm of bioinspired artificial intelligence.

## Materials and Methods

**Figure 1:**
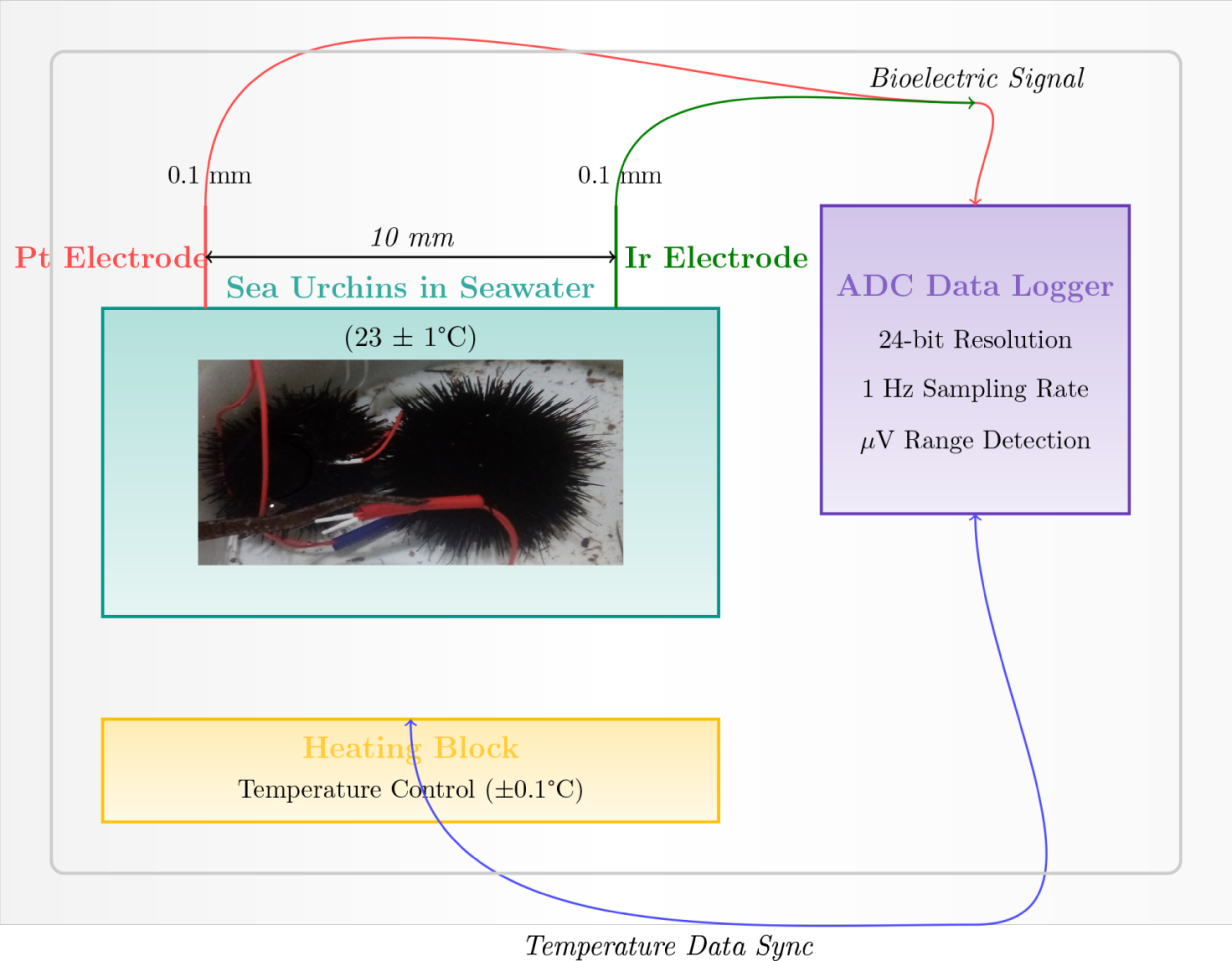
Sea urchin electrochemical characterization setup schematic. A container holds the sea urchins in seawater solution with two needle electrodes (Pt- and Ir-coated stainless steel wires) positioned 10 mm apart. A high-precision 24-bit ADC data recorder captures voltage responses from the electrodes. A heating block beneath the container controls and monitors temperature. The heating block and data logger synchronously register thermal and electrical parameters. This setup enables the detection of minute voltage variations in the μV range, allowing the mapping of spatiotemporal voltage responses in the sea urchin system. Data are sampled at a rate of 1 measurement per second.

The electrochemical behavior of live sea urchins was investigated using a custom-built setup, as illustrated in Figure 1. A rectangular container filled with seawater served as the primary vessel, housing the live sea urchin specimens. Our experiments were initiated on July 14, 2024, at 14:00 local time, and recordings continued for 24 h to specifically capture both daytime and nighttime activity patterns. Three specimens of sea urchins were collected from the coastal area known as “Petalouda” near the city of Patras, Greece. The specimens were found in their natural habitat, concealed among rocks in the littoral zone. Electrophysiological recordings were obtained from the mouthparts (Aristotle’s lantern) of the sea urchins. The Aristotle’s lantern was chosen as the recording site due to its accessibility and dense neural innervation. Two needle electrodes, made of platinum (Pt) and iridium (Ir) coated stainless steel wires (0.1 mm in diameter), were carefully placed within the muscle of the Aristotle’s lantern, about 2 mm apart. The electrodes were inserted to a depth of 1.5 mm to ensure stable contact with the neuromuscular tissue while minimizing damage to the surrounding structures. This allowed for consistent recording of bioelectric activity linked to feeding movements and their neural control. After a 10-min stabilization period, we began recordings. This ensured the signals were normal physiological activity. These electrodes were connected to a high-precision 24-bit analog-to-digital converter (ADC) data recorder, capable of detecting and recording minute voltage fluctuations in the microvolt (μV) range. This sensitivity allowed for detailed mapping of spatiotemporal voltage responses within the sea urchin system. The recordings were done with sea urchins in natural seawater at room temperature (23 ± 1 °C). This provided a stable environment and a constant conductivity medium throughout the experiments. To account for possible artifacts from mechanical movement or changes in media conductivity, we took control recordings from the seawater alone and from immobilized sea urchins. These confirmed that the observed potential changes were due to the organism’s bioelectric activity. The electrodes had a noble-metal coating (Pt/Ir), which ensured minimal interference at the electrode-tissue interface. Their placement within the muscle of the Aristotle’s lantern provided direct access to neuromuscular signals. The surrounding seawater shielded them from electrical noise.

Temperature control and monitoring were achieved through a heating block placed beneath the container. This heating element was synchronized with the data logger, enabling the simultaneous recording of both thermal and electrical parameters. The data acquisition rate was set at 1 sample per second, providing a high-resolution temporal profile of the system’s electrochemical dynamics. This configuration allowed for a detailed examination of the bioelectric activity of sea urchins in relation to different experimental conditions, such as changes in temperature and potential external stimuli.

The recording system had a 1 s temporal resolution. This was to capture the slow bioelectric phenomena of sea urchin nervous systems. These systems work on longer time scales than vertebrate neural signaling. This is due to their unique calcium-based mechanisms.^17^ The slower potentials show coordinated activity in the whole nervous system and not just individual fast action potentials.

The recorded bioelectric activity (*V*_bio_) was measured as the potential difference between two electrodes (Δ*V* = *V*_Pt_ – *V*_Ir_) positioned in the Aristotle’s lantern muscle tissue. The signal acquisition system achieved a voltage resolution of  μV at a sampling rate of *f*_s_ = 1 Hz. Environmental noise was minimized through differential recording, where common-mode signals (*V*_cm_) were rejected according to
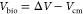
1Control experiments accounted for any thermal effects on the signals. They induced temperature variations (Δ*T* = ±2 °C) while monitoring bioelectric responses. A cross-correlation analysis of the thermal and electrical data showed minimal coupling. This confirmed that the observed bioelectric patterns were not thermally driven.

## Results

**Figure 2:**
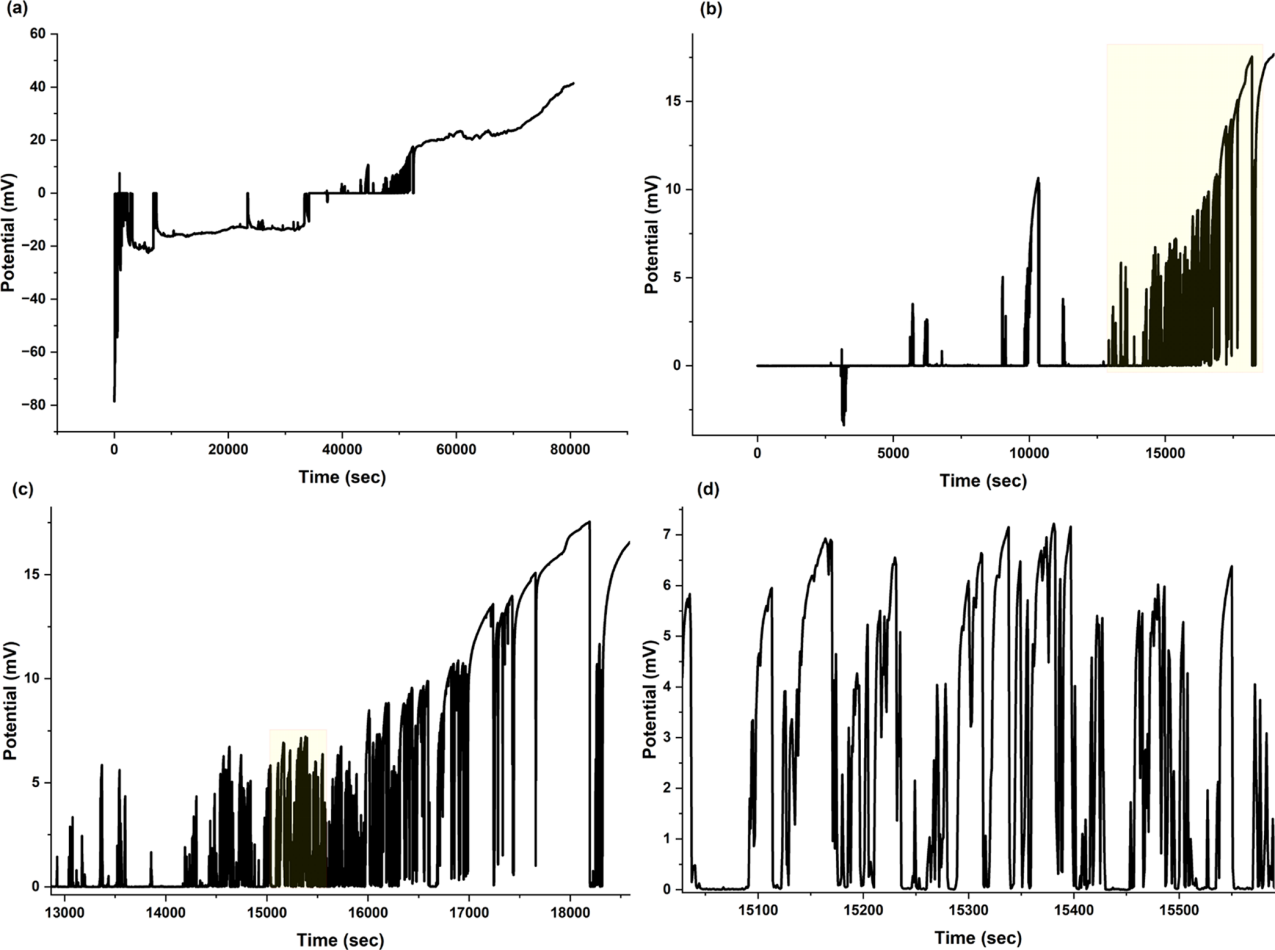
Characteristic bioelectric spiking patterns observed in sea urchins. (a) Representative trace of electrical potential (mV) versus time (s), demonstrating typical spiking activity. The baseline fluctuates around 8.80 mV (median potential), with rapid transient spikes occurring at irregular intervals. (b–d) Examples of large depolarization events: (b) Spikes of pronounced depolarizations reaching the maximum recorded amplitude of 21.05 mV, illustrating the upper range of bioelectric activity. (c) A series of closely spaced large depolarizations, showcasing potential burst-like activity with peak-to-peak distances up to 17.36 mV. (d) Extended depolarization events lasting several seconds, which can indicate prolonged state changes in urchin bioelectric signaling. The lack of negative potentials in (c) and (d) may also be affected by slow baseline drift over time, emphasizing the dynamic nature of sea urchin bioelectric activity. These data highlight the significance of taking into account species-specific factors when analyzing neural signaling patterns.

The electrophysiological recordings obtained from sea urchins demonstrate complex bioelectric activity patterns, indicating advanced information processing ability in these organisms, despite their absence of a centralized nervous system. Figure 2 depicts the distinctive spiking patterns observed in sea urchins. The baseline potential exhibits fluctuations of about 8.80 mV, interrupted by sudden, short-lived spikes that occur at irregular intervals (Figure 2a). The baseline activity observed in the urchin’s distributed neural system may indicate a state of rest, similar to the default mode network found in higher-order organisms. This could suggest a basic level of cognition or awareness of the environment. The significant depolarization episodes depicted in Figure 2b–d are especially noteworthy. The prominent spikes, with amplitudes of up to 21.05 mV, demonstrate the wide range of the urchin’s ability to produce bioelectric signals. The presence of closely spaced massive depolarizations (Figure 2c) resembles the burst firing of neurons in more complex nervous systems, indicating a possible method for amplifying signals or encoding highly significant information. The bioelectric activity patterns seen in Figure 2c,d exhibit a fascinating feature of sea urchin brain signaling: the potential reaches zero but does not fall into negative values. The phenomenon can be explained by various distinctive biological features specific to the neurobiology of echinoderms. Sea urchin neurons are expected to have ionic mechanisms that are different from those found in typical vertebrate neurons.^18^ The ionic mechanisms in sea urchin neurons differ from those in vertebrates. Unlike conventional vertebrate neurons, sea urchin neurons are different. They use unique calcium-dependent action potentials to generate action potentials. The former mainly rely on sodium and potassium channels.^19^ Calcium conductance in sea urchin neurons is crucial for signal propagation. Voltage-gated calcium channels mainly mediate depolarization.^20^ The calcium-based signaling system may explain the recorded potentials. This is true, especially for the long depolarization events in the bioelectric recordings. Additionally, sea urchin neurons exhibit distinct characteristics in their synaptic transmission mechanisms. Sea urchin neuromuscular junctions show strong facilitation and post-tetanic potentiation.^21^ This contrasts sharply with that of the classical vertebrate neuromuscular junction model. The echinoderm nervous system shows plasticity in its ionic responses. Evidence suggests unique chloride conductances affect their firing patterns.^22^ The ionic mechanisms likely evolved to meet the needs of the radially symmetric nervous system. They allowed for local control of the muscle groups. But, they also allowed for coordinated, whole-body responses.^9^

If negative potentials are not present, this could suggest that depolarizing currents are more dominant or that there is a lack of strong hyperpolarizing mechanisms. This leads to spike shapes that are noticeably different from the typical action potentials seen in vertebrates.^23^ Additionally, the signals that were seen could potentially indicate complex electrical potentials originating from many neurons or muscle fibers within the decentralized nervous system of the sea urchin.^24^ This architectural feature has the potential to combine different electrical activities, which could hide individual negative potentials and provide an overall signal profile that is not negative. The sea urchin nervous system, which has evolved to have radial symmetry and distributed control, is decentralized in nature. As a result, it may display unique electrical properties when compared to more centralized nervous systems.^25,26^ This organization has the potential to generate specific spike characteristics that are optimized for local processing and coordination throughout the animal’s body. Additionally, the apparent lack of negative potentials could be influenced by a gradual baseline drift over the course of the recordings. The continuous interaction of sea urchins with their surroundings and the internal changes in their state can lead to gradual variations in the total electrical activity of their nervous system.^21^ This drift has the ability to alter the reference point of the recordings, creating a perception that potentials do not become negative when, in reality, the baseline has undergone a change.^27^

The prolonged depolarization events, lasting several seconds (Figure 2d), are interesting because they could suggest long-lasting variations in the urchin’s bioelectric signaling. The consistent changes in electrical activity may indicate advanced cognitive processes, such as decision-making and memory development, which are usually linked with more sophisticated neural systems.

**Figure 3:**
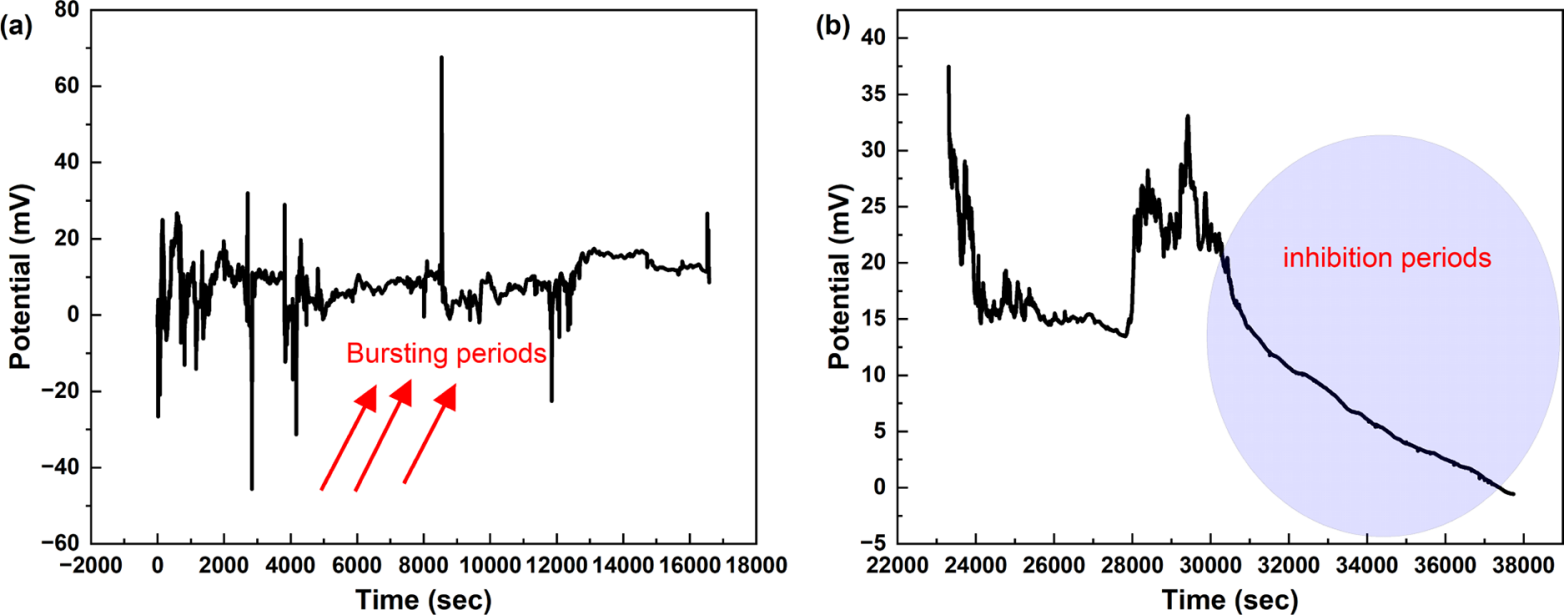
Analysis of firing activity patterns in sea urchin bioelectric signals. (a) A time series depicting changes in membrane potential, consisting of 16,587 data points. The red arrows indicate bursting activity, which is characterized by clusters of fast, high-amplitude spikes. These bursts could indicate episodes of heightened information processing or synchronized responses to signals from the surroundings. (b) The blue circles represent areas with possible inhibitory activity, where the membrane potential decreases below the baseline. This could be due to regulatory mechanisms or sensory adaption processes. The range of reported potential values varies from −45.64 to 67.67 mV. The average potential value is 8.72 mV, with a standard deviation of 5.41 mV. The median potential value is 8.79 mV. The significant range of variation in voltage (113.31 mV) indicates a diverse set of signaling episodes. The complex sequences of neuronal activity, namely, the oscillation between periods of intense firing and periods of inhibition, may be associated with varying levels of consciousness or responsiveness in the sea urchin. Bursting activity may indicate periods of increased sensory processing or decision making, similar to attention-like states observed in more advanced species. On the other hand, inhibitory periods could indicate times of decreased responsiveness or alterations in internal conditions. Although it is not possible to establish a direct link with consciousness, as is understood in more complex organisms, the various firing patterns observed indicate the presence of an advanced information processing system. This system is capable of integrating multiple inputs and producing different outputs, which may support a basic form of awareness that is suited to the sea urchin’s ecological role.

Figure 3 displays interesting patterns in the bioelectric activity of sea urchins, which provide insights into the potential neural-like information processing in these organisms. The detected episodes of bursting activity, indicated by red arrows, signify periods of heightened signaling that may correspond to increased information processing or coordinated reactions to external stimuli. These bursts, which consist of groups of strong and sudden increases in potential activity, are similar to the patterns of nerve cell firing that are linked to states of attention in more advanced organisms.^28^ On the other hand, the inhibitory periods (blue circles) may indicate regulatory mechanisms or sensory adaptation processes, similar to the refractory periods reported in conventional neural networks.^29^ In vertebrate neurons, traditional neural refractory periods are 1–2 ms (absolute) to 10–20 ms (relative).^30^ Even in invertebrates such as Aplysia, refractory periods are under 300 ms.^31^ However, these inhibitory periods in sea urchins last several thousand seconds. They are much longer than the refractory periods in conventional neural networks. Typical neural refractory periods in vertebrates range from milliseconds to seconds.^30^ However, we observed prolonged inhibitory states (about 4000–6000 s, Figure 3b) that suggest a fundamentally different physiological mechanism. These long periods of reduced activity may reflect broader changes in the sea urchin’s distributed nervous system. They may relate to metabolic regulation or adaptation to the environment.^21^ The long duration of these inhibitory periods might be due to unique, calcium-dependent mechanisms in sea urchin neurons.^19^ They could support longer-lasting modulation of neural activity compared to the sodium–potassium systems typical of vertebrate neurons. This distinctive feature of sea urchin neurobiology may reflect an evolutionary adaptation. It lets them control their internal balance for long periods. It also optimizes energy use in response to changing environments.

The extensive range of membrane potentials (−45.64 to 67.67 mV) suggests a diverse set of signaling capacities, which could facilitate complex information processing. The variation in signaling patterns observed here corresponds to the concept of criticality in brain systems, which is a state that maximizes information processing and has been associated with awareness in more advanced organisms.^32^ Although it is too early to attribute human-like consciousness to sea urchins, the many firing patterns seen indicate a complex information processing system that can support a basic kind of awareness suited to the sea urchin’s specific environment.^33^

Sea urchins’ bioelectric activity patterns show features of criticality. This is a state between order and disorder that optimizes information processing. Our data show this. First, the power spectrum analysis reveals a power law decay with a scaling exponent of 6.21 ± 0.06. This is consistent with the critical dynamics observed in other neural systems. Second, the timing of bursting and inhibition shows scale-free patterns. They span multiple time scales (4 to 3102 s). This is a key feature of systems near criticality. Third, the potential values range from −45.64 to 67.67 mV. The complex neuronal activity, with its intense firing and inhibitions, suggests critical dynamics. They may maximize the system’s information processing. The signatures of criticality in sea urchins’ simple nervous system suggest that operating near critical points may be a key principle of neural organization. This may enable even early evolved organisms to process environmental information optimally, despite limited neural resources.

From a biocomputation standpoint, the oscillation between moments of intense activity and periods of suppression offers a fascinating model for the processing of information. This pattern might be regarded as a type of temporal coding, in which information is recorded not only in the frequency of spikes but also in their exact timing and grouping.^34^ This coding technique has the potential to facilitate complex computations using a neural architecture that is relatively simple, which is an appealing characteristic for bioinspired computing systems.^35^

Furthermore, the patterns that have been seen indicate a type of information processing that is spread out, which aligns with the decentralized nervous system found in sea urchins. This distributed architecture shares resemblances with some designs of artificial neural networks, specifically reservoir computing models, in which complex calculations arise from the dynamics of a recurrent network.^36^ The sea urchin’s capacity to produce complex firing patterns with a very rudimentary nervous system provides valuable insights for the advancement of effective, biologically inspired computer architectures.^37^

To summarize, although the bioelectric activity recorded in sea urchins may not directly correspond to higher-order consciousness, it does demonstrate a degree of complexity in information processing that poses a challenge to our understanding of awareness and cognition in basic animals. These findings not only enhance our understanding of echinoderm neurobiology but also provide vital insights for the disciplines of biocomputation and biological intelligence, potentially motivating novel ways of information processing and adaptive behavior in artificial systems.

**Figure 4:**
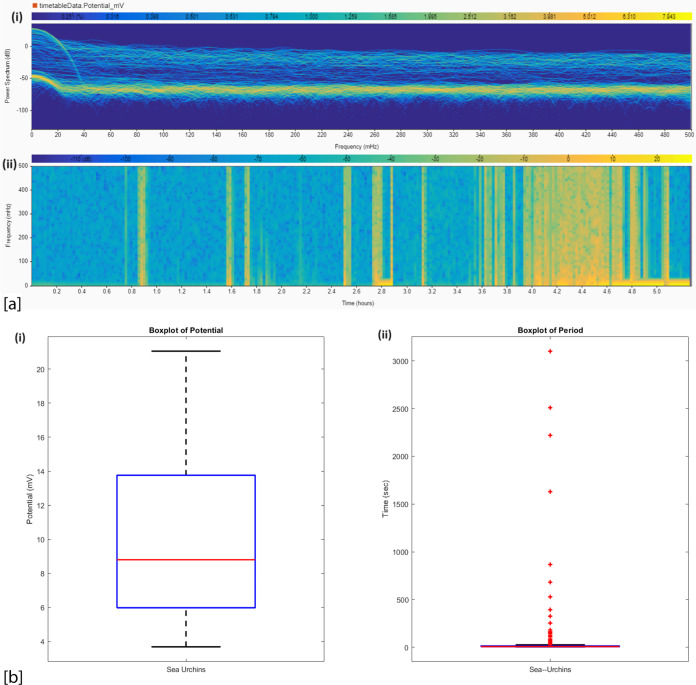
Electrochemical characterization of sea urchin bioelectric activity. (a) Frequency domain analysis: (i) power spectrum in dB versus frequency (mHz), revealing dominant oscillation patterns; (ii) scalogram depicting time–frequency distribution of signal energy, highlighting temporal variations in frequency components. (b) Statistical analysis of potential fluctuations: (i) boxplot of electrical potential (mV) showing median 8.80 mV, interquartile range 5.98–13.77 mV, and outliers (full range: 3.69–21.05 mV, RMS: 11.88 mV); (ii) boxplot of oscillation periods (s) with median 7.00 s, interquartile range 5.00–13.00 s, and extreme outliers extending to 3102.00 s. Note the log scale for period due to high variability (SD: 219.68 s). These analyses reveal complex, multiscale bioelectric dynamics in sea urchins, characterized by both rapid fluctuations and prolonged state changes.

Figure 4a offers an additional understanding of the information processing capacities of sea urchins through frequency domain analysis. The power spectrum displays the most prominent oscillation patterns, but the scalogram emphasizes changes in frequency components over time. The multiscale temporal organization of bioelectric activity in this context is similar to the hierarchical processing observed in vertebrate brains, where different frequency bands are linked to specific cognitive activities.

The statistical examination of potential variations (Figure 4b) highlights the complex structure of sea urchin bioelectricity. The diverse array of measured potentials (ranging from 3.69 to 21.05 mV) and periods (ranging from 4 to 3102 s) indicates a large variety of signaling types. The oscillation periods that deviate significantly from the norm, reaching durations of more than 3000 s, are especially remarkable. These extended alterations in state could indicate large-scale processes of integrating information, possibly similar to slow-wave oscillations linked to memory consolidation in vertebrate brains.

These findings indicate that sea urchins have a very advanced bioelectric signaling mechanism. The observed activity patterns reflect the ability to process information instantly (as shown by the fast spiking behavior) and maintain a state for a long time (as suggested by prolonged depolarizations and long-period oscillations). Although it is not yet appropriate to attribute consciousness to these species in the same manner as it exists in vertebrates, these findings indicate a degree of information processing and interaction with the environment that surpasses basic reflexive behaviors.

The varied and intricate bioelectric activity witnessed in sea urchins poses a challenge to our understanding of the evolution of the nervous system and the fundamental requirements for sophisticated information processing in living organisms. This generates interesting questions about the essence of cognition and consciousness, proposing that these phenomena might occur along a spectrum rather than being binary characteristics of neural systems. Additional investigation of the functional implications of these bioelectric patterns could yield vital insights into the underlying principles of cerebral information processing and the emergence of consciousness.

The power law behavior found in the persistent spectrum of sea urchin bioelectric activity (Figure S1) offers fascinating insights into the dynamics of the system. The spectrum may be accurately characterized by a logarithmic fit equation of the form
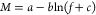
2The scaling exponent, denoted as *b*, is determined to be 6.21 ± 0.06. This value is particularly intriguing within the framework of critical dynamics in biological systems. Bak et al.^38^ found that systems operating at the crucial point between order and chaos frequently display power law scaling in their frequency spectra. The exponent reported is within the typical range associated with critical events in brain systems.^39^ These findings indicate that the bioelectric activity of sea urchins may function at a state of criticality, maintaining an unstable equilibrium between highly organized and chaotic patterns. The concept of criticality has been suggested as a key principle of brain computation, enhancing the efficiency of information processing, flexibility, and dynamic range.^40^ The wide range of spectral distribution, ranging from −91.91 to 10.53 dB, provides additional evidence for this view, suggesting the existence of fluctuations over several scales, which is a characteristic feature of critical systems. Nevertheless, the moderate coefficient of determination (*R*^2^) value of 0.5545 indicates that although the power law model well captures important features of the dynamics, there might be extra complexities that are not completely accounted for by this straightforward scaling relationship. This observation may indicate the distinctive decentralized structure of the sea urchin nervous system, which has the ability to combine various partially independent processing centers.^9^ The potential significance of sea urchin bioelectric activity presents opportunities for gaining deeper knowledge of information processing in primitive organisms and may offer insights into the evolutionary beginnings of complex brain dynamics.

## Discussion

The investigation of bioelectric activity in sea urchins uncovers fascinating similarities with neurological information processing, indicating prospective opportunities for biocomputation and models of information processing that resemble those of the brain. The results of our study reveal complex patterns of neural activity, time-dependent changes, and behaviors resembling those observed in interconnected networks. These findings highlight the need for additional investigations in the field of bioinspired computing.

### Significance of Findings

The spiking behavior observed in sea urchins, which is characterized by varying amplitudes and interspike intervals, shows remarkable similarities to the patterns of neuronal firing in more complex animals.^41^ This implies that even in species without a centralized nervous system, there may be complex methods for processing information. The spike frequency spectrum (Figure S1) has a power-law distribution, which is significant since it corresponds to the concept of criticality in brain systems.^40^
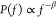
3

Here, *P*(*f*) is the power spectral density, *f* is the frequency, and β is the power-law exponent. In our data, β ≈ 6.2, which falls within the range typically associated with critical dynamics in neural systems.^39^

**Figure 5:**
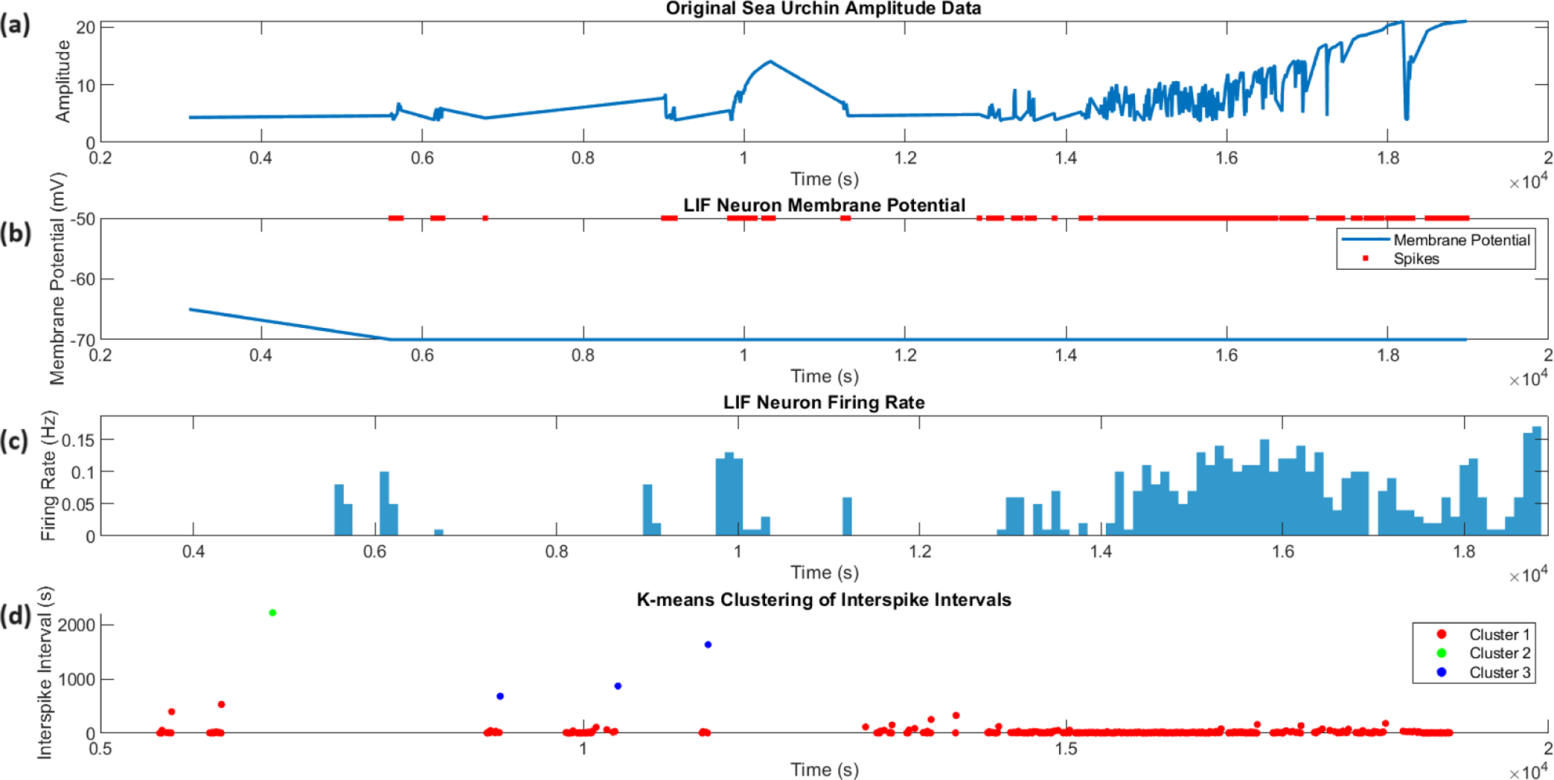
Leaky Integrate-and-Fire (LIF) neuron model applied to sea urchin bioelectric data. (A) Original sea urchin amplitude data over time, showing the raw bioelectric signal. (B) Simulated LIF neuron membrane potential (blue line) with detected spikes (red dots) at the threshold potential. The model generated a total of 516 spikes over the recording period. (C) Calculated firing rate of the LIF neuron model in 100 ms bins. The firing rate varies from 0 to 10 Hz, with an average of 0.03 Hz. The LIF neuron parameters were adapted for echinoderm-like neurons: membrane time constant  ms, resting potential *E*_L_ = −65 mV, membrane resistance *R*_m_ = 100 MΩ, threshold potential *V*_th_ = −50 mV, and reset potential *V*_reset_ = −70 mV. This simulation demonstrates how sea urchin bioelectric signals might be processed in a simplified neural-like system, potentially providing insights into information encoding in these organisms. (D) k-means clustering of interspike intervals (ISIs) revealing three distinct firing patterns: Cluster 1 (red) with short ISIs (mean 15.60 s, *n* = 511), Cluster 2 (green) with a single long ISI (2219.00 s), and Cluster 3 (blue) with medium ISIs (mean 1060.33 s, *n* = 3). LIF neuron parameters were adapted for echinoderm-like neurons: membrane time constant  ms, resting potential *E*_L_ = −65 mV, membrane resistance *R*_m_ = 100 MΩ, threshold potential *V*_th_ = −50 mV, and reset potential *V*_reset_= −70 mV. This simulation demonstrates how sea urchin bioelectric signals might be processed in a simplified neural-like system, revealing complex firing patterns that could represent different functional states or responses to varying inputs.

Our k-means analysis (Figure 5d) has identified unique spike clusters, indicating the presence of multiple functional states or “channels” of information processing. The occurrence of several states in this system is similar to the varied spiking patterns observed in cortical neurons and may serve as the foundation for a basic method of encoding information.^42^

### Comparison with Spiking Neural Networks

The results of our research exhibit significant similarities with artificial spiking neural networks (SNNs) and other bioinspired computing methods. The sea urchins exhibit irregular spiking patterns and variable interspike intervals, which bear resemblance to the behavior of leaky integrate-and-fire (LIF) neurons. LIF neurons are a widely used model in spiking neural networks (SNNs).^43^ The equation that governs the membrane potential in a LIF (leaky integrate-and-fire) neuron is
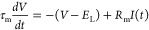
4

where *V* is the membrane potential, τ_m_ is the membrane time constant, *E*_L_ is the resting potential, *R*_m_ is the membrane resistance, and *I*(*t*) is the input current.

The observed criticality in sea urchin bioelectric activity correlates with current research on critical dynamics in spiking neural networks (SNNs), which has demonstrated that networks operating at the boundary between order and chaos have excellent information processing capabilities.^32^ This implies that sea urchins may have adapted to function in a comparable critical state, maintaining an unstable balance between stability and flexibility in their information processing.

Figure 5 illustrates the application of a Leaky Integrate-and-Fire (LIF) neuron model to sea urchin bioelectric data. The simulation results provide intriguing insights into potential neural-like processing in these organisms. The LIF model, using parameters adapted for echinoderm-like neurons, generated 516 spikes over the recording period, with firing rates ranging from 0 to 10 Hz and an average of 0.03 Hz. These values are consistent with the relatively slow neural dynamics observed in echinoderms, as reported by Bullock and Horridge^44^ The sporadic high-frequency bursts (up to 10 Hz) amidst generally low firing rates align with observations by Cobb and Laverack^45^ who noted that echinoderm neurons can exhibit both tonic and phasic firing patterns.

The variable amplitude of the original signal (Figure 5A) and the resulting nonuniform firing pattern (Figure 5B,C) suggest a complex encoding of information, possibly reflecting the decentralized nervous system of sea urchins.^24^ This complexity may be related to the integration of various sensory inputs and motor outputs, as sea urchins lack a centralized brain but possess a distributed nervous system capable of coordinating behaviors.^46^ The observed firing patterns could represent a form of temporal coding, where information is encoded in the timing of spikes rather than just their rate, a phenomenon observed in other invertebrate systems.^47^

Interestingly, the average firing rate of 0.03 Hz is lower than what is typically observed in vertebrate neurons, but it is not unprecedented in slow-conducting invertebrate nervous systems.^48^ This low rate might be indicative of energy-efficient information processing, a critical adaptation for organisms in variable marine environments.^49^ Furthermore, the extensive variation in firing rates (ranging from 0 to 10 Hz) indicates that sea urchin neural-like cells have the potential to encode a wide variety of stimulus intensities or types. This aligns with their need to react to various environmental signals.^50^

While this LIF model provides valuable insights, it is important to note that actual sea urchin neurons may exhibit more complex dynamics not captured by this simplified model. For instance, Cobb and Laverack^45^ observed nonlinear responses in echinoderm neurons that may not be fully represented in a LIF model. Future work could explore more sophisticated models, such as Hodgkin–Huxley-type models, to better capture the nuances of echinoderm neural dynamics.

### Implications for Novel Computing Paradigms

The bioelectric activity patterns identified in sea urchins present fascinating opportunities for the advancement of innovative computer architectures. The decentralized nature of information processing in these organisms, without a central brain, serves as a model for highly parallel and resilient computation.^37^ This has the potential to stimulate the development of novel designs for edge computing or sensor networks, where distributed and energy-efficient processing is of the utmost importance.

The observed significance in the bioelectric dynamics of sea urchins indicates possible uses in reservoir computing, a field where systems operating on the boundary of chaos have demonstrated exceptional ability in handling time-based information.^51^ By replicating the self-organized criticality observed in sea urchins, we could potentially create reservoir computing systems that are more effective and adaptable.

Moreover, the diverse behavior revealed in our spike sorting analysis has the potential to contribute to the advancement of multivalued logic systems, surpassing the limitations of conventional binary computing.^52^ These devices have the potential to provide higher computing density and potentially more energy-efficient information processing.

### Potential Link to Consciousness

Although it is too early to attribute consciousness to sea urchins in the same manner as to more complex species, our discoveries do inspire thought-provoking questions regarding the basic necessities for awareness or sensibility. The complex temporal patterns and apparent assimilation of information found in sea urchin bioelectric activity exhibit certain similarities with the neuronal characteristics of awareness in more sophisticated brains.^53^

**Figure 6:**
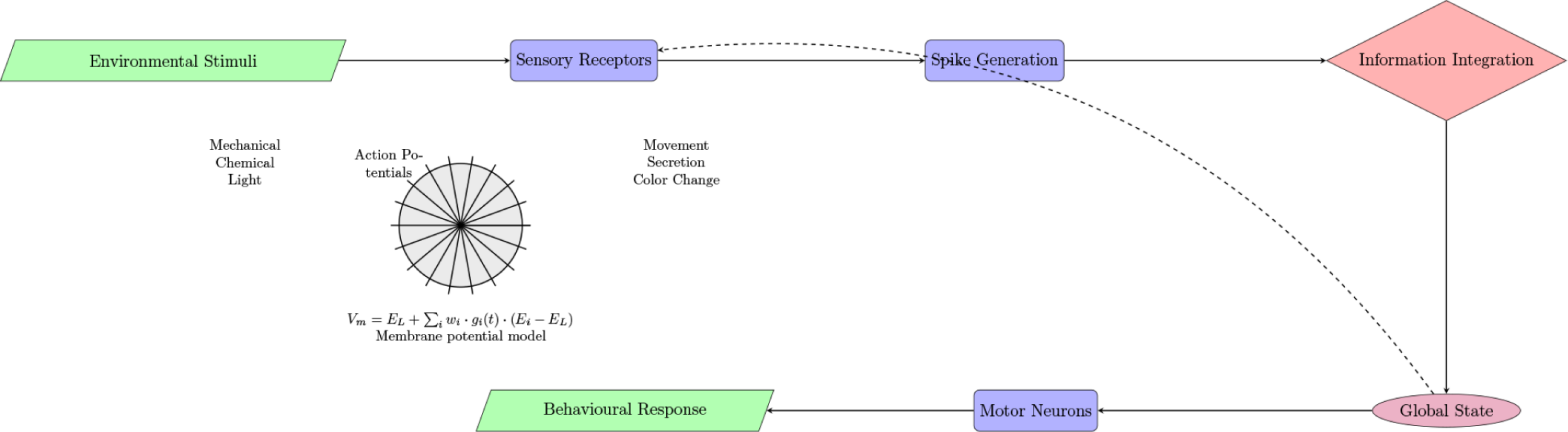
Proposed mechanism of spike-based information processing in sea urchins and its potential correlation to fundamental consciousness. The graphic depicts the progression of information from external stimuli to sensory receptors, the generation of electrical impulses, and their integration, resulting in a collective state that impacts behavior. The dashed line signifies potential feedback mechanisms. For perspective, a simplified outline of a sea urchin and a fundamental equation for membrane potential are provided.

Figure 6 illustrates a proposed mechanism for how spiking activity in sea urchins might relate to a basic form of awareness or responsiveness to the environment.

This model proposes that the incorporation of spiking activity throughout the sea urchin’s decentralized nervous system may lead to a composite state that impacts both sensory processing and motor output, creating a fundamental sensorimotor loop that can be viewed as a preliminary stage for deeper forms of consciousness.

### Limitations and Future Directions

Although our study offers useful insights into the bioelectric activity of sea urchins, future research should address many limitations. Initially, our recordings were restricted to a limited number of sites on the organism. Future research ought to try to simultaneously record data from numerous sites in order to have a deeper understanding of the spatial dynamics of information processing.

Furthermore, the connection between the observed bioelectric activity and particular behaviors or external factors has yet to be fully understood. Conducting controlled trials that establish a correlation between spiking patterns and specific stimuli or behaviors would be essential for accurately understanding these signals.

Future research directions could include:

1 Development of more sophisticated computational models that capture the unique characteristics of sea urchin bioelectric activity.
2 Investigation of the molecular and cellular mechanisms underlying the observed spiking behavior.
3 Comparative studies across different echinoderm species to understand the evolution of these information processing mechanisms.
4 Exploration of potential applications in unconventional computing, such as biohybrid systems that incorporate living sea urchin tissue as computational elements.

## Conclusion

Our study concludes that sea urchins display complex bioelectric activity resembling that of brain systems, which poses a challenge to our understanding of information processing in apparently uncomplicated species. These findings not only enhance our understanding of echinoderm biology but also present interesting opportunities for bioinspired computer architecture. As we further explore the complexities of information processing in nature, we may uncover novel concepts that have the potential to completely transform our methods of computation and biological intelligence.

## Supporting Information Available

Figure S1: Power-law behaviour in the persistent spectrum of sea urchin bioelectric activity. The outer plot shows the FFT Analysis in Log–Log scale, demonstrating the power-law relationship across a broader frequency range. The graph illustrates the relationship between magnitude (M) in decibels (dB) and frequency (f) in Hertz (Hz) in the persistent spectrum, demonstrating a power law decay where spectral magnitude decreases with increasing frequency. The data (grey points) are fitted with a logarithmic model (red curve) using the Log3P1 equation: *M* = *a* – *b* ln(*f* + *c*) where *M* is the magnitude (dB), *f* is the frequency (Hz), and *a*, *b*, and *c* are the fitting parameters. Fitting parameters: *a* = −53.06 ± 0.11, *b* = 6.21 ± 0.06, *c* = (−3.07 ± 1.86) × 10^–5^. The fit quality is indicated by *R*^2^ = 0.5545 and reduced χ^2^ = 31.03. The spectrum spans a magnitude range from −91.91 dB to 10.53 dB, with a mean of −42.53 ± 8.36 dB (*N* = 9492). This broad spectral distribution suggests complex, multi-scale dynamics in sea urchin bioelectric signaling, potentially reflecting diverse information processing mechanisms across different temporal scales (PDF)
