# Supplementary material for: On Oscillations in the External Electrical Potential of Sea Urchins
Source: ACS Omega. 2025 Jan 8;10(2):2327–37. doi: 10.1021/acsomega.4c10277 (PMC11755143; doi:10.1021/acsomega.4c10277)
Supplement: Supplementary file 1 — ao4c10277_si_001.pdf [file ao4c10277_si_001.pdf]

Supplementary Material

On Oscillations of External Electrical Potential of Sea Urchin

Panagiotis Mougkogiannis[1,*] and Andrew Adamatzky[1]

[1]Unconventional Computing Laboratory, University of the West of England, Bristol, UK

Email: Panagiotis.Mougkogiannis@uwe.ac.uk

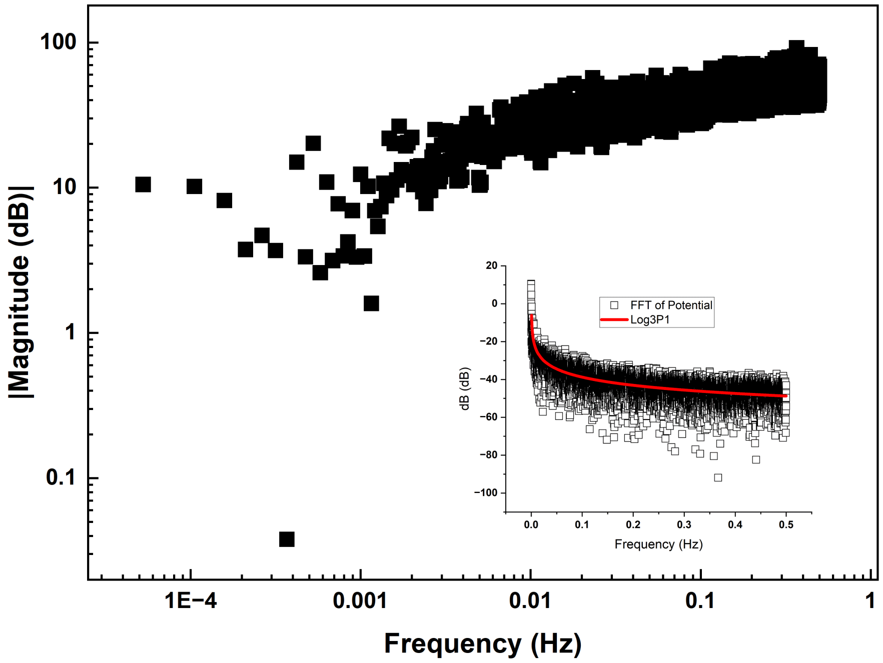

Figure S1: Power law behaviour in the persistent spectrum of sea urchin bioelectric activity. The outer plot shows the FFT Analysis in Log-Log scale, demonstrating the power-law relationship across a broader frequency range. The graph illustrates the relationship between magnitude (M) in decibels (dB) and frequency (f) in Hertz (Hz) in the persistent spectrum, demonstrating a power law decay where spectral magnitude decreases with increasing frequency. The data (grey points) are fitted with a logarithmic model (red curve) using the Log3P1 equation: $M = a - b\ln(f + c)$ where M is magnitude (dB), f is frequency (Hz), and a, b, and c are fitting parameters. Fitting parameters: $a = -53.06 \pm 0.11$, $b = 6.21 \pm 0.06$, $c = (-3.07 \pm 1.86) \times 10^{-5}$. The fit quality is indicated by $R^2 = 0.5545$ and reduced $\chi^2 = 31.03$. The spectrum spans a magnitude range from -91.91 dB to 10.53 dB, with a mean of -42.53 ± 8.36 dB ($N = 9492$). This broad spectral distribution suggests complex, multi-scale dynamics in sea urchin bioelectric signaling, potentially reflecting diverse information processing mechanisms across different temporal scales.

.
